# A Systematic Study of Mechanism of *Sargentodoxa cuneata* and *Patrinia scabiosifolia* Against Pelvic Inflammatory Disease With Dampness-Heat Stasis Syndrome via Network Pharmacology Approach

**DOI:** 10.3389/fphar.2020.582520

**Published:** 2020-12-04

**Authors:** Luanqian Hu, Yuqi Chen, Tingting Chen, Dan Huang, Shihua Li, Shuna Cui

**Affiliations:** ^1^ Jiangsu Key Laboratory of Integrated Traditional Chinese and Western Medicine for Prevention and Treatment of Senile Diseases, Medical College of Yangzhou University, Yangzhou, China; ^2^ Department of Gynecology and Obstetrics, Affiliated Hospital of Yangzhou University, Yangzhou, China; ^3^ Jiangsu Co-innovation Center for Prevention and Control of Important Animal Infectious Diseases and Zoonoses, College of Veterinary Medicine, Yangzhou, China

**Keywords:** pelvic inflammatory disease, dampness-heat stasis syndrome, network pharmacology, mechanism, *Patrinia scabiosifolia*, *Sargentodoxa cuneata*

## Abstract

**Objective:** To investigate the mechanism of *Sargentodoxa cuneata* (Oliv.) Rehder & E.H.Wilson (*SC*) and *Patrinia scabiosifolia* (*PS*) against Pelvic Inflammatory Disease with Dampness-Heat Stasis Syndrome via network pharmacological approach and experimental validation.

**Methods:** The active compounds with OB ≥ 30% and DL ≥ 0.18 were obtained from TCMSP database and further confirmed by literature research. The targets of the compounds and disease were acquired from multiple databases, such as GeneCards, CTD and TCMSP database. The intersection targets were identified by Venny software. Cytoscape 3.7.0 was employed to construct the protein-protein interaction (PPI) network and compound-target network. Moreover, GO enrichment and KEGG pathway analysis were analyzed by DAVID database. Finally, CCK-8, Griess assay and a cytometric bead array (CBA) immunoassay were used for experimental validation by detecting the influence of the active compounds on proliferation of macrophage, release of NO and TNF-α after LPS treatment.

**Results:** 9 bioactive compounds were identified from *SC* and *PS*. Those compounds corresponded to 134 targets of pelvic inflammatory disease with dampness-heat stasis syndrome. The targets include vascular endothelial growth factor A (VEGFA), von willebrand factor (VWF), interleukin 6 (IL6), tumor necrosis factor (TNF) and nuclear transcription factor 1 (NFκB1). They act on the signaling pathways like advanced glycation end products-receptor of advanced glycation end products (AGE-RAGE), focal adhesion (FA), Toll-like receptor (TLR) and nuclear transcription factor κB (NF-κB). In addition, by *in vitro* validation, the selected active components of *SC* and *PS* such as acacetin, kaempferol, linarin, isovitexin, sinoacutine could significantly inhibit the release of NO induced by LPS, respectively. Moreover, different dose of acacetin, kaempferol, isovitexin and sinoacutine significantly inhibits the TNF-α production.

**Conclusion:** This study provides solid evidence for the anti-inflammatory mechanism of *SC* and *PS* against pelvic inflammatory disease with dampness-heat stasis syndrome, which will provide a preliminary evidence and novelty ideas for future research on the two herbs.

## Introduction

Pelvic inflammatory disease is a common gynecological disease caused by lower reproductive tract infection, bad menstrual hygiene and postoperative infection. The main clinical symptoms is the change of menstrual volume, abnormal increase of leucorrhea, menstrual extension and abdominal pain (Liu et al., 2019). The acute pelvic inflammatory disease further causes tubal ovarian cyst, tubal obstruction, which may lead to infertility, ectopic pregnancy, chronic pelvic pain and so on. Recently, studies have shown that this disease is closely linked with the occurrence of ovarian tumor (Stewart et al., 2020). Moreover, recurrent chronic pelvic inflammatory disease not only affects female reproductive health, but also enhances family and socioeconomic burden. Antibiotic treatment is widely used in clinical practice, however, the long medication cycle is highly associated with drug resistance (Dodson, 1994). Thus, it is urgent to develop effective therapeutic approach against chronic pelvic inflammatory disease.

Treatment according to syndrome differentiation is one of the major feature in Traditional Chinese Medicine, which is well correlated with the concept of personalized medicine by tailoring of medical treatment to the individual characteristics of each patient. Traditional Chinese Medicine classifies pelvic inflammatory disease as lesser-abdominal pain, leukorrhagia, abdominal mass. In the TCM theory, “Downward flow of damp and heat” is the main syndrome of pelvic inflammatory disease (Liu et al., 2019).Therefore, the main treatment principle of pelvic inflammatory disease is heat-clearing and detoxicating, transforming stasis and removing blood, eliminating dampness and softening hardness (Ma et al., 2017). The combination of antibiotic and herbal medicine in the clinical practice significantly improve the symptoms and reduce the recurrence rate. However, the underlying molecular mechanism of this treatment is not yet known (Zhang et al., 2018c).

**FIGURE 1:**
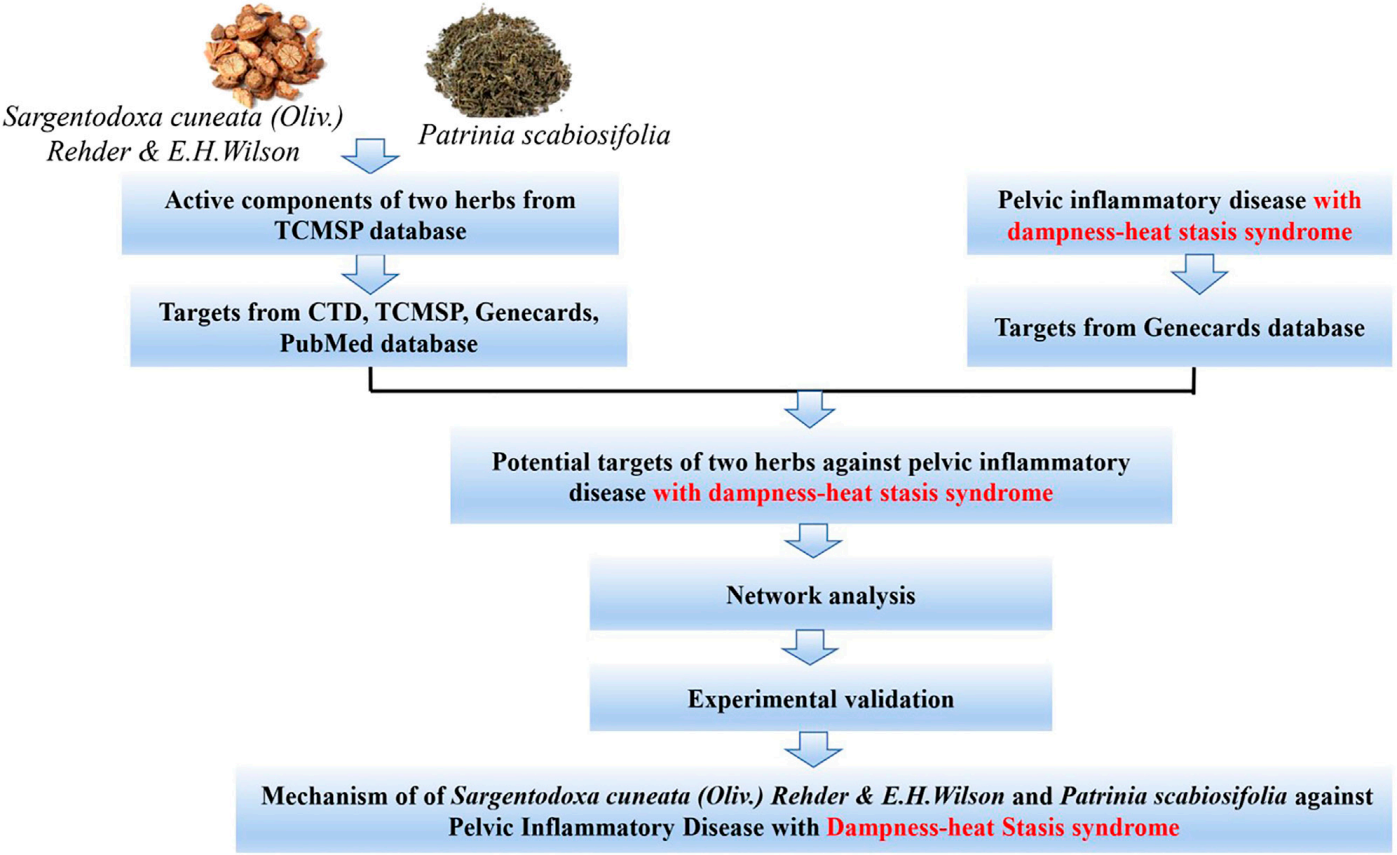
Flowchart of designed analysis in *SC* and *PS* against pelvic inflammatory disease with dampness-heat stasis syndrome.

*Sargentodoxa cuneata* *(Oliv.) Rehder *& *E.H.Wilson* (*SC*) was firstly recorded in *Ben Cao Tu Jing* with the property of heat-clearing and detoxicating, blood quickening, dispelling wind (Xu et al., 2017). *Patrinia scabiosifolia* (*PS*) was firstly recorded in *Shen Nong Ben Cao Jing* featured by heat-clearing and detoxicating, dispersing abscess and expelling pus, transforming stasis and relieving pain. In China*, SC* and *PS* are widely used in clinical treatment of pelvic inflammatory disease, ulcerative colitis, appendicitis, prostatitis and other inflammatory diseases. Recently, UPLC-QTOF-MS/MS or GC/MS studies have shown that both SC and PS contains a variety of active components such as phenols, flavonoids, phenylpropenoids and triterpenoids, which exerts anti-oxidant, anti-bacteria, anti-inflammatory and anti-virus effects (Lee et al., 2012; Hwang et al., 2019; Yang et al., 2019). A large number of experimental data showed that herbal extracts and natural products were effective in preventing and treating several disease such as Non-alcoholic fatty liver disease, fungal infection (Tocci et al., 2018; Zhang et al., 2018b). However, due to the complexity of the composition of the herbs, the underlying molecular mechanism of this two herbs in treatment of pelvic inflammatory disease is not clear. Therefore, the worldwide clinically application is significantly limited. Although the investigation based on “Disease-Syndrome-Formula-Targets” is quite challenging, Network pharmacology approach is a useful tool to decipher the complexity of Chinese herbs by building multiple components-targets-disease network (Liu et al., 2015). Therefore, in this study, network pharmacology approach and experimental validation methods were used to clarify the mechanism of this two herbs in treatment of pelvic inflammatory disease with dampness-heat stasis syndrome. The results will provide the scientific evidence to explain the state of the art of “clearing heat and transforming dampness” theory of TCM in the treatment of pelvic inflammatory disease with dampness-heat stasis syndrome. The entire design of this study was showed in the flowchart (Fig. 1).

## Materials and Methods

### Screening the Active Components of *Sargentodoxa cuneata* and *Patrinia scabiosifolia*

The active compounds of *SC* and *PS* were screened with OB ≥ 30% and DL ≥ 0.18 from TCMSP database. TCMSP (http://www.tcmspw.com/tcmsp.php) is a traditional Chinese medicine database and analysis platform based on the pharmacology framework of traditional Chinese medicine system. It provides 12 pharmacodynamics characteristics for drug screening and evaluation, including bioavailability, drug similarity, half-life, caco-2 permeability, blood-brain barrier, etc. In addition, TCMSP also provides diseases targets and drug targets of each active compound and automatically construct a network between them which is useful to reveal the mechanism of traditional Chinese medicine (Ru et al., 2014).

### Collecting Disease Targets

The keywords “pelvic inflammation” or “pelvic inflammatory disease” was inquired from PubMed database (https://www.ncbi.nlm.nih.gov/pubmed/). Then the keywords were introduced to the GeneCards database (https://www.genecards.org/) to search the disease targets. According to the *guideline principle of Chinese traditional medicine new drug clinical research*, the main symptoms of pelvic inflammatory disease with dampness-heat stasis syndrome are “hypogastric pain,” “more leucorrhoea,” “yellow and thick leucorrhoea,” “lumbosacral pain,” “petechias in the tongue,” “thick and greasy tongue coating,” “wiry pulse.” Then we introduced these keywords to GeneCards database. The obtained symptom targets are reflected with the targets of pelvic inflammation. The overlapped targets were considered to be the potential targets of pelvic inflammatory disease with damp-heat stasis syndrome.

### Predicting Targets of *Sargentodoxa cuneata* and *Patrinia scabiosifolia*

Compounds exert their effects by binding to specific molecular targets and regulating their biological activity in transcriptional and protein levels. The collection of compound targets were essential for the study of the chemical-target interaction in order to elucidate the mechanism of compound action. The compound targets are mainly obtained from the following approaches: retrieve compound and collate their targets in CTD database (http://ctdbase.org/), in TCMSP database (http://www.tcmspw.com/tcmsp.php) and in Genecards database (https://www.genecards.org/) as well as in PubMed database (https://www.ncbi.nlm.nih.gov/pubmed/). The Uniprot database (http://www.uniprot.org/) is used for gene name standardization. In total, compound targets of *SC* and *PS* were identified after removing the duplicate targets.

### Predicting Compounds-Disease Targets

These drug targets and disease targets were introduced into Venny2.1.0 software (http://bioinfogp.cnb.csic.es/tools/venny/) to draw the Venn diagram. The overlapped targets were considered to be potential therapeutic targets of *SC* and *PS* against pelvic inflammatory disease with dampness-heat stasis syndrome.

### Constructing Protein-Protein Interaction Network

The PPI network interaction was obtained by introducing these potential therapeutic targets of *SC* and *PS* to STRING database (https://string-db.org) with the species limited to “Homo sapiens” and a confidence score > 0.9. The results were further visualized by Cytoscape3.7.0. The degree of freedom was reflected by the node size and color. Subsequently, the top 15 key targets were ranked by MCC method in Cytohubba.

### Constructing Component-Target Network

The targets of each component were compared with the targets of pelvic inflammatory disease with damp-heat stasis syndrome to obtain the compound- disease target. Then, those targets were introduced into Cytoscape 3.7.0 to construct the component-target network. In the network, edges represented interactions between nodes, nodes represented compounds and targets.

### Analyzing GO Enrichment and KEGG Pathway

DAVID Bioinformatics resources 6.7 database (https://www.david.ncifcrf.gov/) was selected for Gene Ontology enrichment analysis of the biological processes. *p* value was used to evaluate the protein existing in each GO annotation, reflecting the significance of the biological function. In addition, FDR error control method (FDR < 0.05) was used to test and correct the *p* value. The threshold value *p* < 0.05 was finally set to screen the biological processes with significant differences. Eventually, the molecular function (MF), biological process (BP) and cellular component (CC) of the target gene or protein were enriched for gene function analysis. The pathway visualization enrichment analysis was carried out on the top 20 pathways by R package.

## Experimental Verification

### Reagents Source

Acacetin, Kaempferol, Linarin, Isovitexin and Sinoacutine were purchased from Selleck Company (Shanghai, China). The detailed information of the compounds was listed in Table 1. CCK-8 kit was purchased from Beyotime Biotechnology (Shanghai, China); *p*-aminobenzene sulfonic acid, sodium nitrite and n-(1-naphthyl)-ethylenediamine dihydrochloride were purchased from China National Pharmaceutical Group Corporation (Shanghai, China). Mouse TNF CBA Flex set C8 was purchased from BD Pharmingen (Becton Dickinson, San Diego, CA).

**TABLE 1:** Information of the compounds for experimental validation.

Compound	Chemical formula	Molecular weight	OB (%)	DL	Concentration
Acacetin	C_16_H_12_O_5_	284.28	34.97	0.24	12.5–100 μM
Kaempferol	C_15_H_10_O_6_	286.25	41.88	0.24	12.5–100 μM
Linarin	C_28_H_32_O_14_	592.6	39.84	0.71	4–32 μM
Isovitexin	C_21_H_20_O_10_	432.41	31.29	0.72	25–100 μM
Sinoacutine	C_19_H_21_NO_4_	325.39	63.39	0.53	37.5–100 μM

### CCK-8 Assay

Macrophage RAW264.7 cell line was purchased from Cell Bank of Shanghai (Institutes of Life Sciences, Chinese Academy of Science). The cells were grown in incubator with 37°C and 5% CO_2_. Cell viability assay was performed according to the previously reported (Cui et al., 2020). RAW264.7 cells were seeded into 96-well plates with the density of 1 × 10^6^/ml, and then stimulated with or without LPS together with different concentrations of compounds. After 24 h incubation, CCK-8 was added in the dark and incubated about 1h after gently mixing on a constant temperature shaker. The plate was then measured on a microplate reader (BioRad, USA) with the OD 450 nm.

### Nitrite Oxide Detection

The experiment was done following with the previous literature (Cui et al., 2020). The cells were prepared according to the cell viability assay. After 20 h incubation, 50 μL of the supernatant was taken from each well and then 50 μL Griess reagent (Griess A:Griess B = 1:1) was added to each well. After 10 min incubation, the plate was measured on a microplate reader (BioRad, USA) with OD 540 nm. The concentration of nitrite in the supernatant was calculated by the standard curve.

### Cytometric bead Array Immunoassay for TNF-α Detection

The experiment was done following with the previous literature (Jiménez et al., 2005). 1 × 10^5^ cells/ml were treated with LPS together with different concentration of compounds for 2 h, respectively. The supernatant were collected and measured by CBA immunoassay. The procedure was followed by the manufacture’s protocol. Briefly: 50 μl of sample or standard of cytokine were added to 50 μl of Capture Beads and incubated for 1 h, respectively. 50 μl of PE Detection Reagent were added to the mixture, further incubated in darkness at room temperature for 1 h, and then washed before data acquisition by flow cytometry. The concentration of TNF-α in the supernatant was calculated by the standard curve.

### Data Analysis

Data were expressed as mean ± standard deviation (*X*¯ ± SD). SPSS 21.0 software was used to analyze the data. Student’s-t test was used for comparison between groups. And *p* < 0.05 was considered as statistically significant.

## Results

### Acquiring and Screening the Chemical Component

A total of 16 candidate components of *SC* and *PS* were screened from TCMSP according to oral availability (OB) ≥ 30% and druglikeness (DL) ≥ 0.18. The components with undiscovered targets were excluded. Totally, 9 kinds of active ingredients were obtained, with 3 compounds from *SC* and 8 compounds from *PS.* The basic information of the compounds was shown in Table 2.

**TABLE 2:** Active compounds of *SC* and *PS*.

Serial number	Herbs	Active component	Mol ID	OB (%)	DL
1	*SC*	Catechin	MOL000096	49.68	0.24
2	*PS*	Bolusanthol B	MOL001678	39.94	0.41
3	*PS*	Acacetin	MOL001689	34.97	0.24
4	*PS*	Kaempferol	MOL000422	41.88	0.24
5	*PS*	Stigmasterol	MOL000449	43.83	0.76
6	*PS*	Luteolin	MOL000006	36.16	0.25
7	*PS*	Quercetin	MOL000098	46.43	0.28
8	Shared	Beta-sitosterol	MOL000358	36.91	0.75
9	Shared	Sitosterol	MOL000359	36.91	0.75

### Integrating Disease-Component Target

**FIGURE 2:**
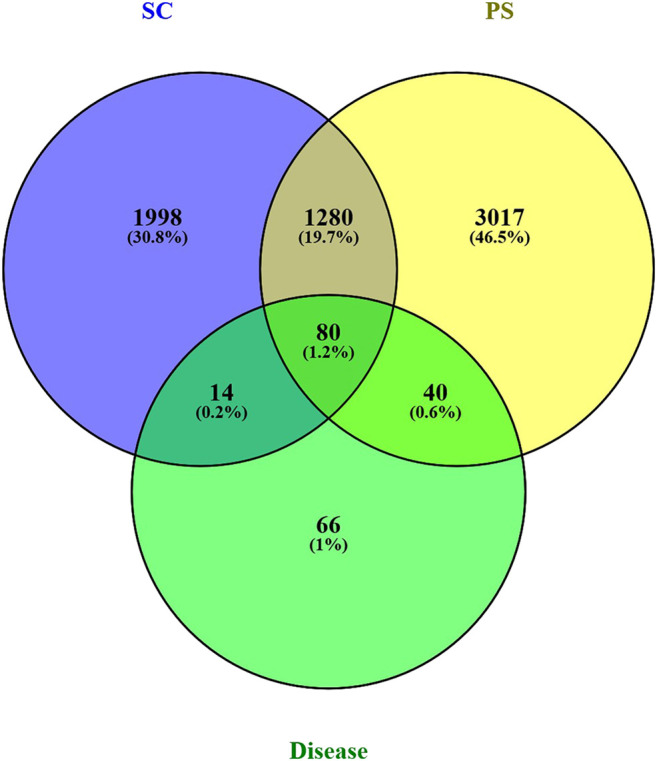
Venn’s diagram of targets of compound-disease. Blue circle represents targets of *SC*; yellow circle represents targets of *PS*; green circle represents targets of pelvic inflammatory disease with dampness-heat stasis syndrome. The overlapped 134 genes were the potential therapeutic targets of two herbs for the disease.

2294 genes of pelvic inflammatory disease and 300 genes of the syndrome of dampness-heat stasis were screened from Genecards database. After integrating, we eventually obtained 200 genes of the pelvic inflammatory disease with dampness-heat stasis syndrome. Then, CTD, TCMSP, Genecards and Uniprot databases were used to identify the targets, respectively. The number of the targets were listed: catechin: 3,159; Acacetin: 70; Kaempferol: 289; stigmasterol: 82; Luteolin: 294; quercetin: 3,443; beta-sitosterol: 106; sitosterol: 107; bolusanthol B: 26. Eventually, the component targets and disease targets were introduced into Venny2.1 to draw venny plot. It clearly showed that 134 genes were the potential therapeutic targets of *SC* and *PS* for the treatment of pelvic inflammatory disease with dampness-heat stasis syndrome (Figure 2).

### Constructing Protein-Protein Interaction Network

**FIGURE 3:**
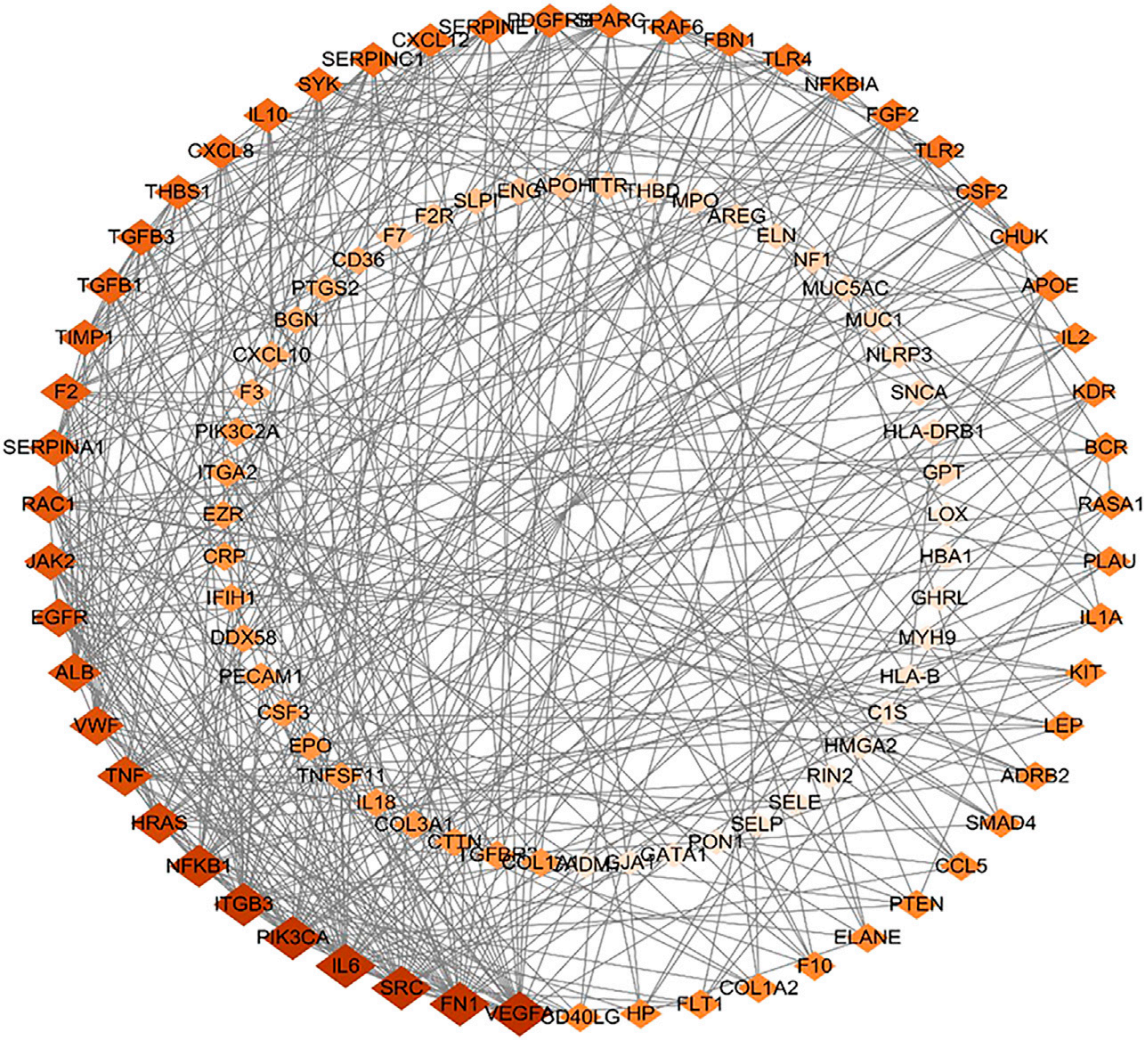
The protein-protein interaction (PPI) network of core targets of *SC* and *PS* in the treatment of pelvic inflammatory disease with dampness-heat stasis syndrome. The network included 106 nodes and 427 edges, 51 targets with degree 0–5 in the inner circle and 55 targets with degree 6–26 in the outer circle. (Diamonds represent the targets, color from dark to light indicates decreasing importance).

**FIGURE 4:**
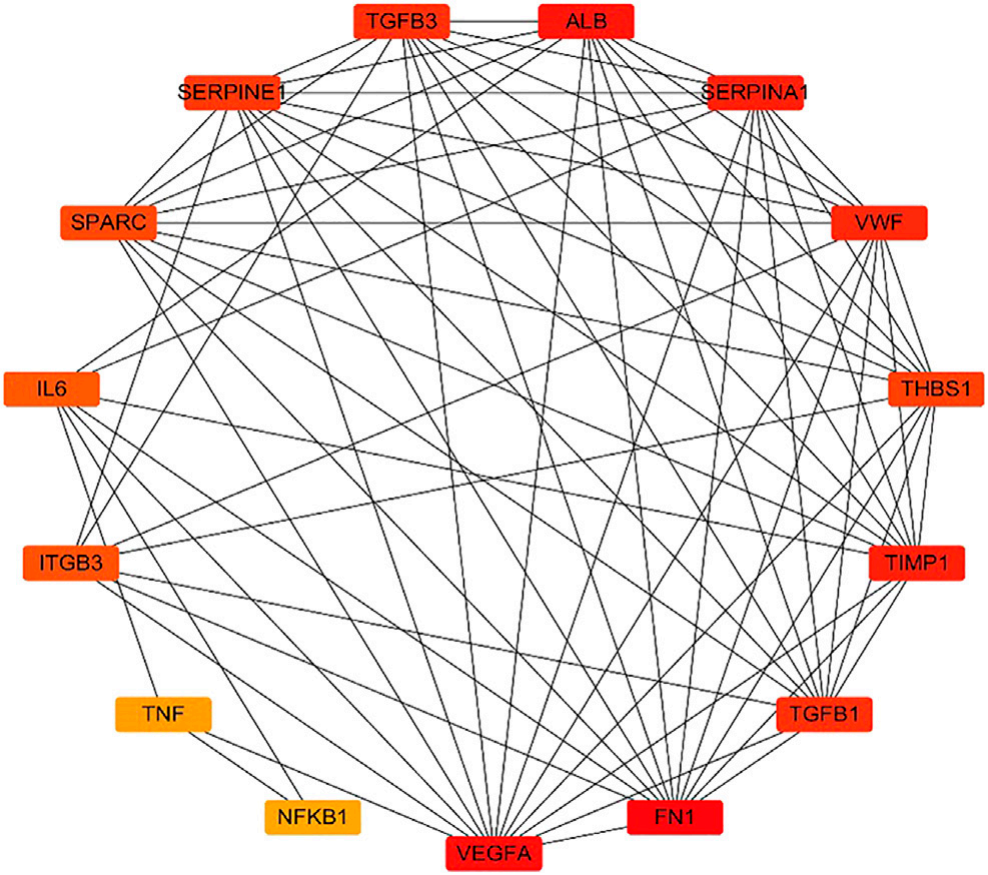
Analysis of the top 15 hub targets network of *SC* and *PS* in the treatment of pelvic inflammatory disease with dampness-heat stasis syndrome ranked by MCC method. (Rectangles represent the targets, color from red to yellow indicates decreasing importance).

Further, 134 potential therapeutic targets were imported into STRING database. Then the results screened with the criteria of confidence > 0.9 were recorded into Cytoscape3.7.0 to construct PPI network. In the network, the node represents the target gene, the size and color of the node represents the degree of freedom. As shown in Figure 3, the network was consisted of 106 nodes and 427 edges, 51 targets with degree 0–5 in the inner circle and 55 targets with degree 6–26 in the outer circle. Then, the top 15 key targets were ranked by MCC method in CytoHubba. As shown in Figure 4, the key target proteins for the *SC* and *PS* against pelvic inflammatory disease with dampness-heat stasis syndrome included vascular endothelial growth factor A (VEGFA), von willebrand factor (VWF), interleukin 6 (IL6), tumor necrosis factor (TNF) and nuclear transcription factor 1 (NFκB1).

### Constructing Component-Target Network

**FIGURE 5:**
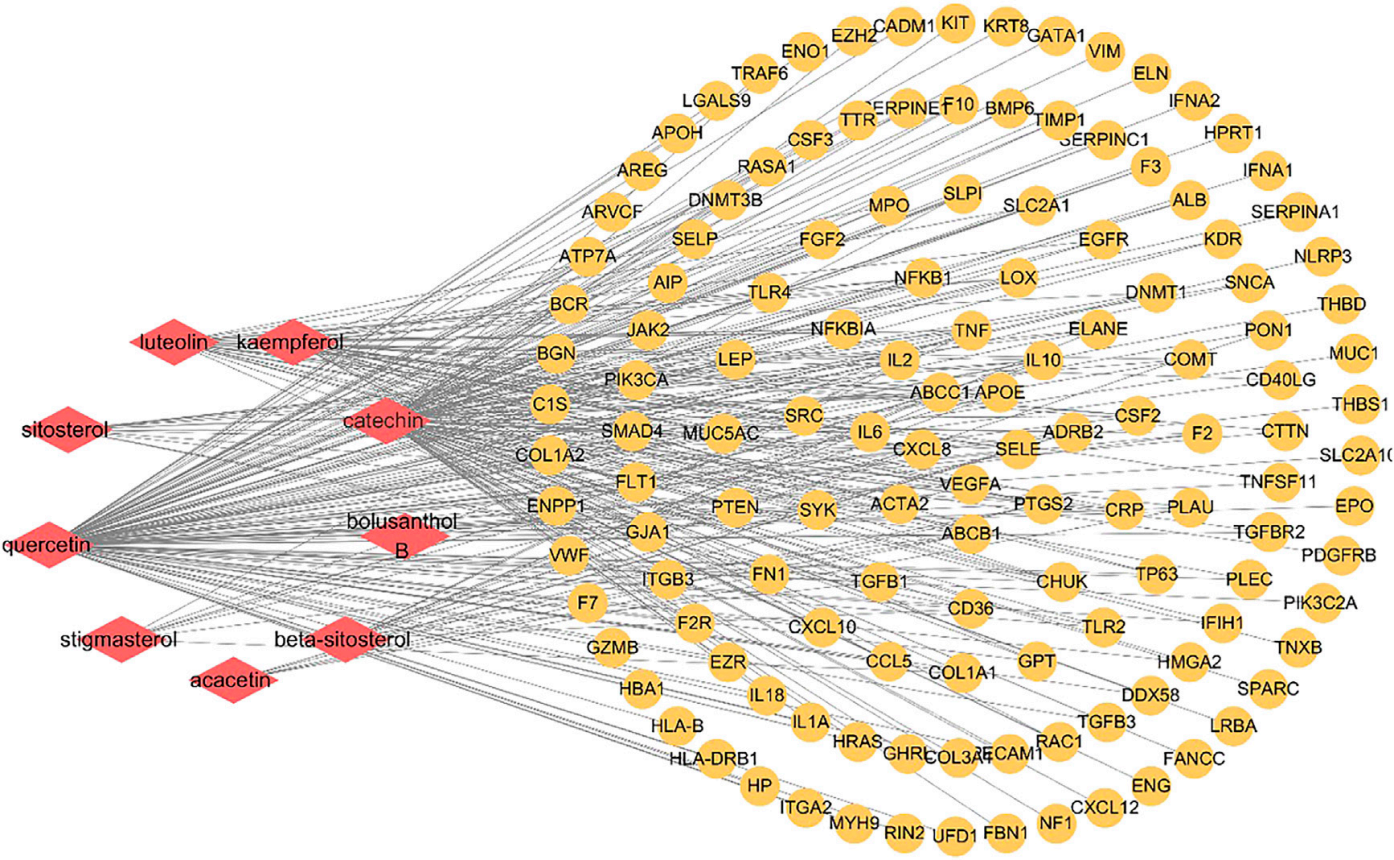
Component-target network of *SC* and *PS* in the treatment of pelvic inflammatory disease with dampness-heat stasis syndrome. The network included 9 active components and 134 key targets. (Diamonds represent compounds, circles represent targets).

9 active components and 134 key targets of *SC* and *PS* were imported into Cytoscape3.7.0 to construct component-target network. As shown in Figure 5, 143 nodes and 308 edges were included in this network. The diamond represented the compound, the circle represented the target, and the edge represented the correlation. By deeply analysis of network, the result showed that quercetin, catechin, luteolin and kaempferol were the main components of the anti-inflammatory action of *SC* and *PS.* Multiple targets corresponded to the same active components, conversely, one target could also correspond to different components. The results fully verified that *SC* and *PS* against pelvic inflammatory disease with dampness-heat stasis syndrome were based on the multi-compounds, multi-targets and multi-pathways mechanism.

### GO Enrichment Analysis

**FIGURE 6:**
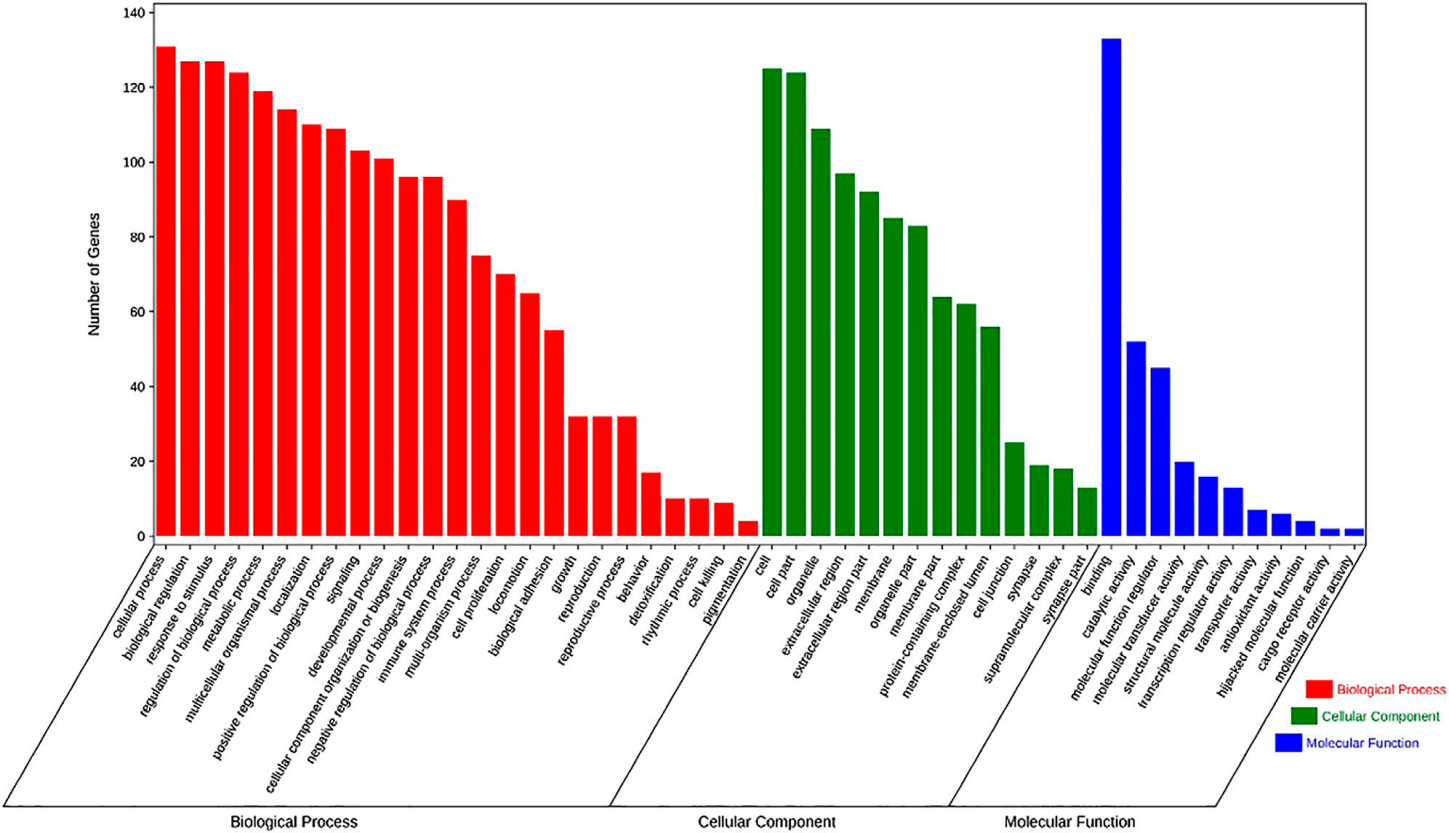
The enrichment analysis in biological processes, cellular components and molecular functions of 134 identified targets of *SC* and *PS*, with 25 in biological processes, 14 in cell components and 11 in molecular functions.

In order to clarify the functional distribution of anti-inflammatory targets of SC and PS, 134 predicted targets were imported into DAVID 6.7 database for GO enrichment analysis and KEGG pathway annotation. Basing on the criteria of *p* < 0.05 and FDR < 0.05, those genes were involved in 25 biological processes, 14 cell components and 11 molecular functions. The biological processes were mainly involved in immune system process, cell proliferation, biological adhesion and metabolic process. Cell components included organelle and synapse. Molecular functions of those genes were involved in antioxidant activity, molecular carrier activity, catalytic activity, etc. The detailed information was shown in Figure 6.

### KEGG Pathway Annotation

**FIGURE 7:**
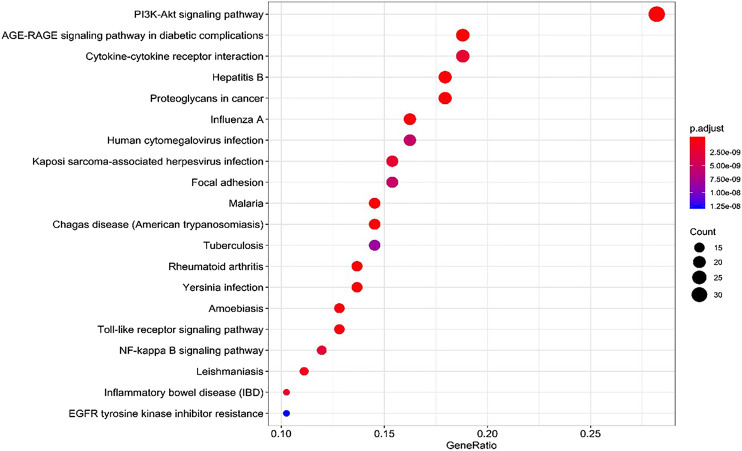
KEGG pathway analysis for the molecular signal pathway of *SC* and *PS* in the treatment of pelvic inflammatory disease with dampness-heat stasis syndrome.

134 potential targets were further introduced into DAVID database for KEGG pathway enrichment analysis. The results showed that these targets were significantly enriched in 85 pathways (*p* < 0.05). As shown in Figure 7, the top 20 pathways were visually analyzed by R package. Among them, advanced glycosylated end product-glycosylated end product receptor (AGE-RAGE), phosphatidylinositol 3 kinase/protein kinase (PI3K/Akt), focal adhesion (FA), Toll-like receptor (TLR), nuclear transcription factor (NF-κB) and some infectious disease such as hepatitis B, malaria, tuberculosis, human cytomegalovivirus infection, cancer pathways as well as cell apoptosis pathways were highly involved. Thus, it can be predicted that the active components of SC and PS played a therapeutic role by regulating the anti-inflammatory related pathways.

### Experimental Validation

We further validated the predicted results by *in vitro* experiment. The active compounds were chosen based on the previously study and literature mining. As acacetin, kaempferol, quercetin are predicted as active compounds by *in silico* study, we previously reported that quercetin inhibited LPS induced macrophage migration by inhibiting iNOS/FAK/Paxillin pathway (Cui et al., 2019). Therefore, acacetin, kaempferol are selected for the further validation. Moreover, the compounds such as Sinoacutine, Isovitexin and Linarin are within the criteria of oral availability (OB) ≥ 30% and druglikeness (DL) ≥ 0.18, but they are not well investigated for the anti-inflammatory effect. Finally, we chose those five compounds Acacetin, Kaempferol, Sinoacutine, Isovitexin and Linarin for further experimental validation.

**FIGURE 8:**
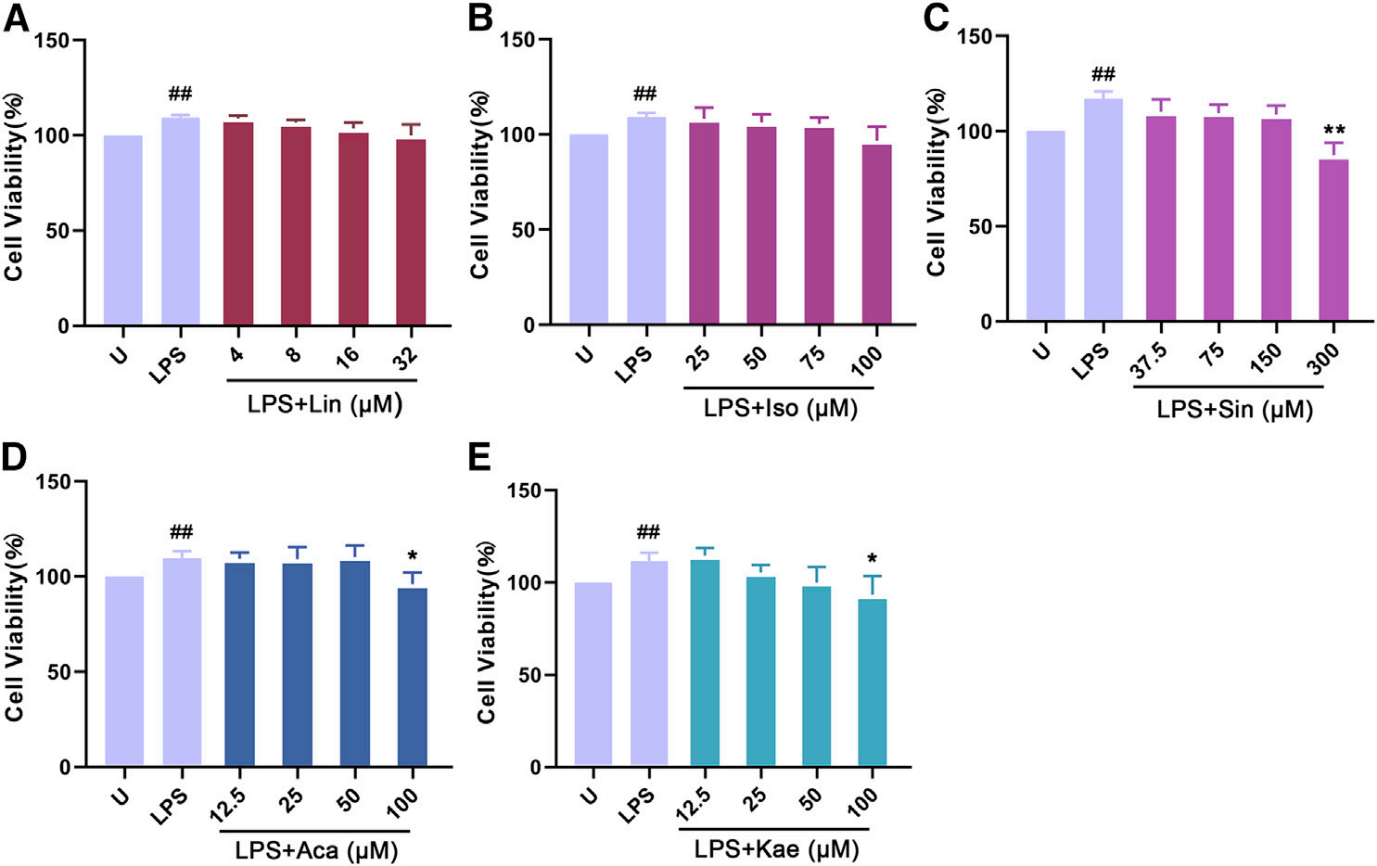
Effects of the active compounds of *SC* and *PS* on cell viability of macrophage induced by LPS. Macrophage was treated for 24 h in the presence of indicated concentrations of **(A)**: Linarin; **(B)**: Isovitexin; **(C)**: Sinoacutine; **(D)**: Acacetin; **(E)**: Kaempferol. (# # represents P < 0.01 compared to the Untreated group; * represents P < 0.05 compared to the LPS group; ** represents P < 0.01 compared to the LPS group).

**FIGURE 9:**
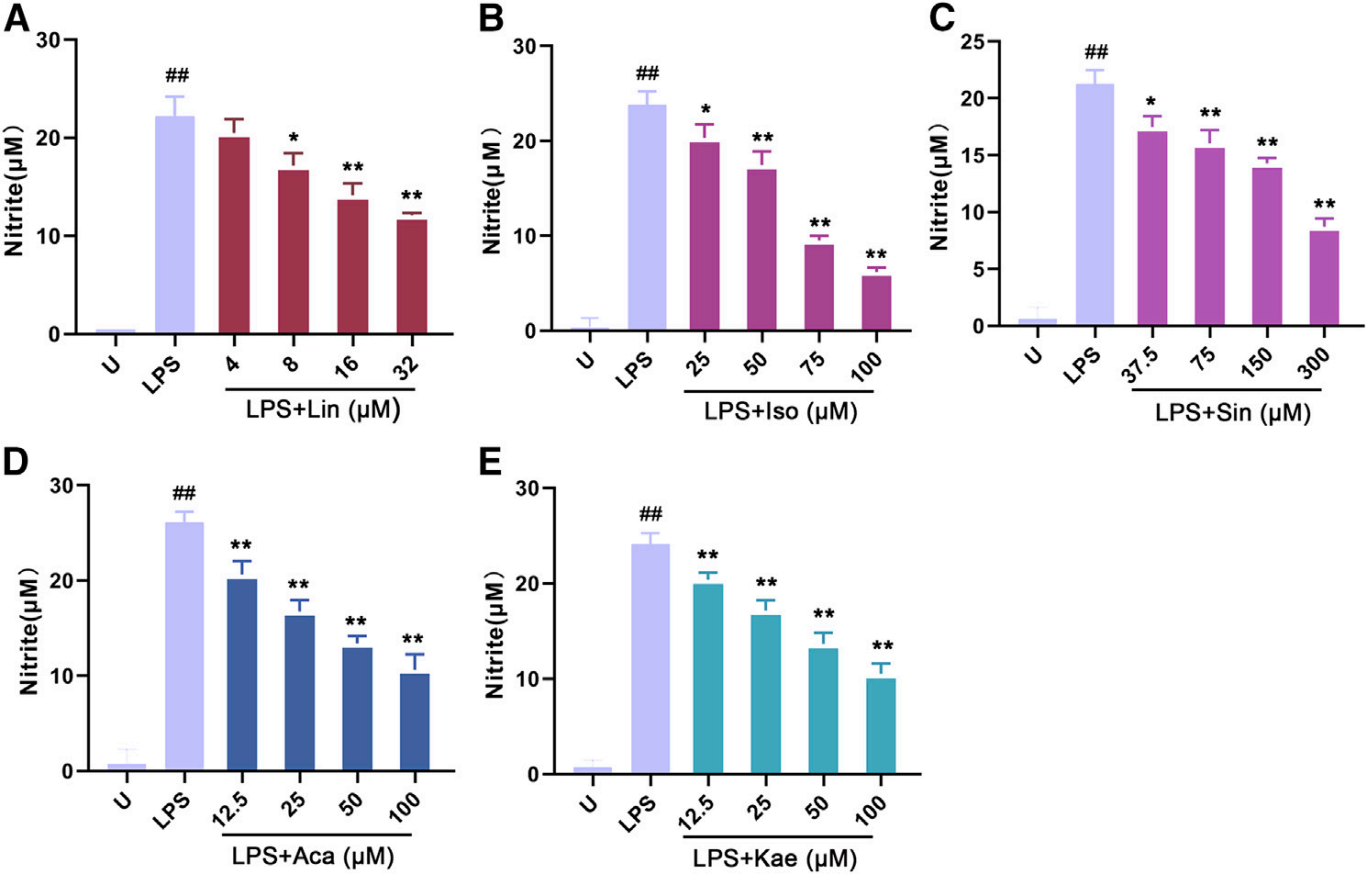
Effects of the active compounds of *SC* and *PS* on release NO of macrophage induced by LPS. Macrophage was treated for 20 h in the presence of indicated concentrations of **(A)**: Linarin; **(B)**: Isovitexin; **(C)**: Sinoacutine; **(D)**: Acacetin; **(E)**: Kaempferol. (# # represents P < 0.01 compared to the Untreated group; * represents P < 0.05 compared to the LPS group; ** represents P < 0.01 compared to the LPS group).

As shown in Figure 8, LPS alone could significantly promote the proliferation of macrophages (*p* < 0.01). When adding different concentration of compounds, this effects were significantly inhibited. The results showed that 300 µM of Sinoacutine, 100 µM of Acacetin and 100 µM of Kaempferol significantly inhibited macrophages proliferation stimulated by LPS. The different concentration of the compounds [4–32 µM of Linarin (A), 25–100 µM of Isovitexin (B), 37.5–300 µM of Sinoacutine (C), 12.5–50 µM of Acacetin (D), and 12.5–50 µM of Kaempferol (E)] showed no significant cytotoxicity (*p* > 0.05). Moreover, we detected the effects of these compound on the NO release after LPS treatment. As shown in Figure 9, the untreated macrophages did not secrete NO. While the NO production in LPS stimulated macrophages were significantly increased (*p* < 0.01). However, different concentration of compounds (8–32 µM of Linarin, 25–100 µM of Isovitexin, 37.5–300 µM of Sinoacutine, 12.5–100 µM of Acacetin and 12.5–100 µM of Kaempferol) can significantly inhibited the release of NO in a concentration-dependent manner. This inhibitory effects on NO production was not due to the cytotoxicity, which indicating that these five compounds showed a strong inhibition of NO production in macrophages.

**FIGURE 10:**
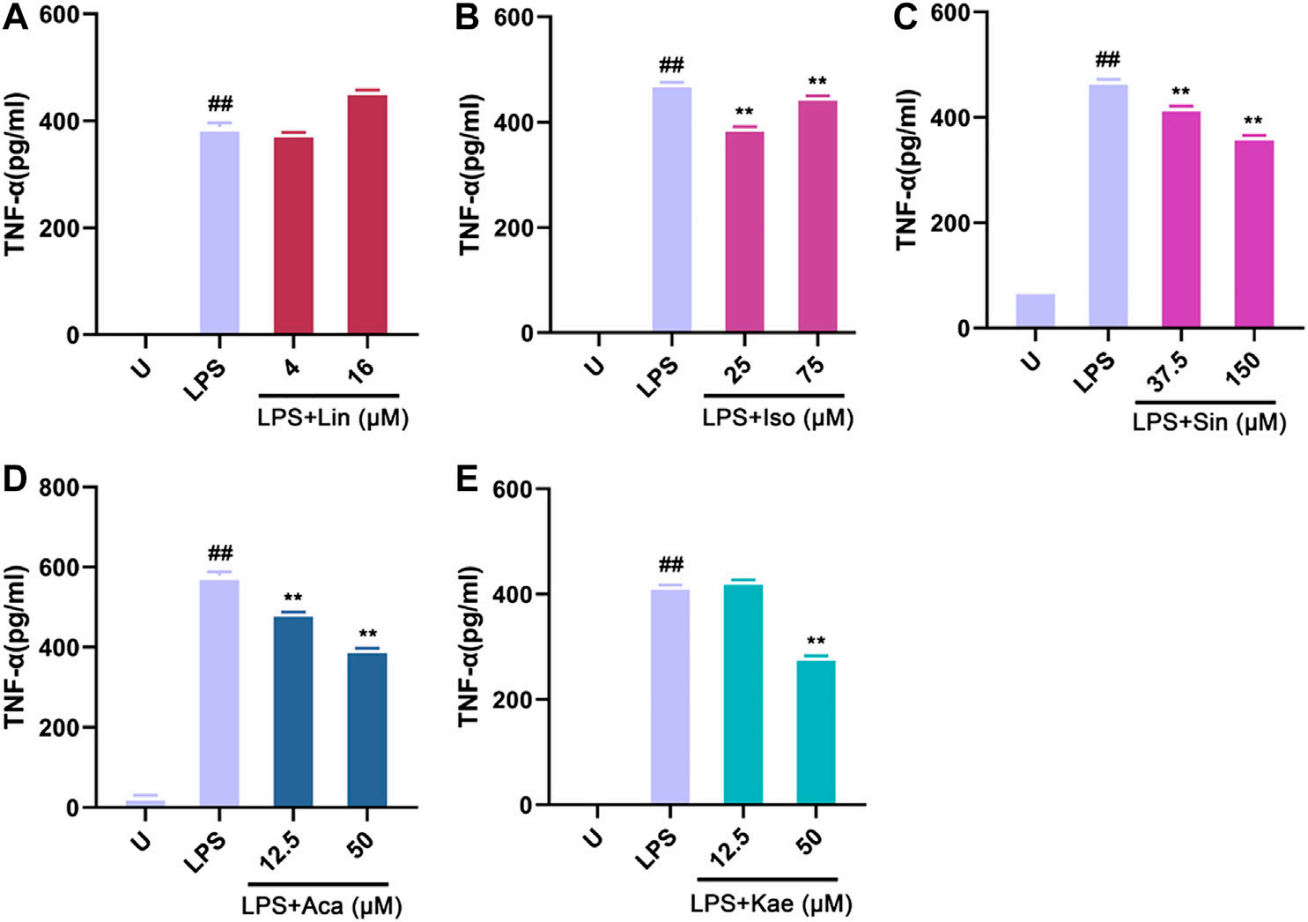
Effects of the active compounds of SC and PS on TNF-α release of macrophage induced by LPS. Macrophage was treated for 2 h in the presence of LPS and indicated concentrations of **(A)**: Linarin; **(B)**: Isovitexin; **(C)**: Sinoacutine; **(D)**: Acacetin; **(E)**: Kaempferol (# # represents P < 0.01 compared to the Untreated group; * represents P < 0.05 compared to the LPS group; ** represents P < 0.01 compared to the LPS group).

Moreover, we investigated the rapid response of the different compound on the release of TNF-α upon LPS stimulation for 2 h. The concentration range of different compounds were selected based on the results of cell viability and NO determination. As shown in Figure 10, the untreated macrophages did not produce the cytokine TNF-α; After LPS stimulation for 2 h, the cells showed a pronounced TNF-α production. The production of TNF-α was significantly decreased by different concentration of compounds: 25–75 µM of Isovitexin, 12.5–50 µM of Acacetin, 37.5–150 µM of Sinoacutine and 50 µM of Kaempferol. However, the effect of Linarin was not significant in this short time period treatment. The results demonstrated that the active components such as Isovitexin, acacetin, Sinoacutine and Kaempferol exerted anti-inflammatory effect against LPS stimulation.

## Discussion

In our study, we showed a novel mechanism of the *SC* and *PS* against pelvic inflammatory disease with dampness-heat stasis syndrome by network pharmacology and experimental validation. The results showed that there were 9 active components acting on key targets such as VEGFA, VWF, IL6, TNF, NFKB1, respectively, as well as regulating signaling pathways such as AGE-RAGE, FA, Toll-like receptor, PI3K/Akt, NF-κB, apoptosis and cancer signaling pathways.

VEGFA, also known as vascular permeability factor, is a member of the platelet-derived growth factor/vascular endothelial growth factor (PDGF/VEGF) family (Liu et al., 2016). It induces the proliferation and migration of vascular endothelial cells. Its expression is also associated with tumor progression and microvascular lesions. The combination of VEGFA and VEGFR2 activates the phosphatidylinositol-3 kinase/protein kinase (PI3K/Akt) signaling pathway (Huang et al., 2017). PI3K/Akt participates in endothelial cell proliferation, vascular permeability, vascular inflammation infiltration and metastasis by regulating the downstream target molecules (Hung et al., 2017). Therefore, the development of relevant inhibitors has great significance in anti-angiogenesis. Recent years, a number of studies have shown that Traditional Chinese Medicine can regulate VEGF in multi-targets and multi-pathways (Qin et al., 2018; Zhang et al., 2018a). Studies have found that the proliferation of vascular endothelial cells was significantly inhibited by leech and cantharides through reducing the expression of VEGF and matrix metalloproteinase 9 (MMP-9) (Yao et al., 2020). *Salvia Miltiorrhiza* and *puerarin* improved the thrombus status by regulating the level of VEGF and the release of coagulation factors (Hu et al., 2020). In a rat model of hepatopulmonary syndrome, quercetin predominantly inhibited pulmonary vascular angiogenesis by regulate VEGFA/VEGFR-2 pathways (Li et al., 2016). Moreover, acacetin played an anti-inflammatory role by regulating the VEGFA (Li and Ren, 2019). Taken together, combined with the literature and the predicted targets via network pharmacology, we predict that the function of promoting blood circulation and removing blood stasis of *SC* and *PS* was finalized by the regulation of the related targets of VEGF. VWF is an important hemostatic protein and the hydrolyzed production is involved in the adhesion of platelets to the site of vascular injury and the transport of proteins in blood (Braun et al., 2020). Hemostasis is the key in the treatment of pelvic inflammatory disease (Graesslin et al., 2019). Quercetin, one of the most abundant secondary metabolites in *PS* was confirmed to predominantly improve the platelet function in rats with cholestatic liver injury by up-regulating the expression of VWF, Ca^2+^ and ORAI1 (Nassef, 2019).

NFκB1 (NF-κB P50) is a member of NF-κB gene family, including P50, p52, RelA (p65), RelB, and c-Rel, which can regulate the expression of a series of genes like cell proliferation, differentiation and apoptosis (Chebotareva et al., 2020). Many extracellular stimulators such as lipopolysaccharides, viruses and inflammatory factors can activate NF-κB. Phosphorylated IκB is covalently bound to ubiquitin molecules. Then its conformation is changed and it is degraded by protease (May and Ghosh et al., 2018; Flohé et al., 1997). NF-κB moves into the nucleus and binds to the promoter sequence in the target gene to induce cell factors such as TNF, IL6, IL-1β, IL-2 as well as the transcription of inflammatory enzymes such as nitric oxide synthnase (NOS) and cyclooxygenase 2 (COX2) (Taniguchi and Karin, 2018). Therefore, NF-κB is the main activator of chronic inflammatory diseases and it is also the key bridge for the transformation of chronic inflammation to tumor. Kaempferol, a dietary element and an important bioflavonoid in vegetables and fruits, has a variety of pharmacological effects via regulating the signaling pathways and inhibiting the expression of transcription factors (Parveen et al., 2007). Kaempferol inhibited the phosphorylation of IκBα and NF-κB p65 and blocked the secretion of downstream inflammatory factors with a slightly dose-dependent manner in rat osteoarthritis chondrocytes (Zhuang et al., 2017).

In addition, both IL-6 and TNF are pro-inflammatory cytokines, which can further stimulate the release of inflammatory mediators and cause tissue damage. In the process of inflammation, IL-6 can not only activate the neutrophils, but also delay the phagocytosis of neutrophils, aggravating the production of inflammatory mediators (Tanaka et al., 2014). It is reported that IL6 can also induce signal transduction as well as phosphorylation of transcriptional activator 3 (STAT3) and protein tyrosine kinase 2 (JAK2) by activating the IL6/JAK2/STAT3 signal pathway (Wang et al., 2018). Then it changes the biological characteristics of cells in the inflammatory environment and eventually differentiated into tumor cells abnormally. TNF acts as an inflammatory mediator, including TNF-α secreted by macrophages and TNF-β secreted by T lymphocytes (Jiao et al., 2020). It is often used as an indicator of some infectious diseases. TNF-α can improve the ability of neutrophils to adhere to endothelial cells to stimulate the local inflammatory response (Wang et al., 2017). Studies have found isovitexin exerted anti-inflammatory on LPS-induced acute lung injury by inhibiting MAPK and NF-κB signaling pathways (Lv et al., 2016). Luteolin inhibited the phosphorylation of IκB and P65 to interrupt the NF-κB signaling pathway in the chronic pharyngitis model (Chen et al., 2018). Moreover, our results showed that Isovitexin, Acacetin, Kaempferol and Sinoacutine all exerts a rapid effect against LPS induced inflammation. However, the effect of Linarin was not pronounced. The results confirmed the anti-inflammatory effect of SC and PC.

Recent studies have found that AGE-RAGE pathway was tightly linked with inflammation (Bierhaus et al., 2006; Yan et al., 2009). NF-kB activated by AGE-RAGE pathway promotes the secretion of inflammatory factors such as NO, IL-1β, TNF-α, IL6 and importantly of RAGE itself, which triggers a positive-feedback loop in AGE-RAGE pathway, aggravating the inflammatory response(Kierdorf and Fritz, 2013). Accumulating evidence indicates that RAGE and TLRs share common ligands and signaling pathways, especially in inflammation (Ibrahim et al., 2013). In chronic inflammatory state associated with diabetes, it was confirmed that high mobility group box 1 (HMGB1) protein, as an activator of TLR and RAGE, promoted the secretion of inflammatory factors (Machado et al., 2011). Researchers found that kaempferol inhibited the release of HMGB1 to cut off AGE-RAGE pathway, alleviating inflammatory response (Kim et al., 2012).

Toll-like receptors (TLRs) are the body's first line of defense against infection due to their quickly recognition and response to the pathogens. Studies on TLRs mediated inflammatory response mainly focus on TLR4. However, recent studies have found that TLR3, which initially appeared as an antiviral agent, can also amplify the inflammatory response via positive feedback regulation. The combination of pathogen’s RNA with TLR will activate MAPK, NF-κB signaling pathways to release inflammatory factor and type I interferon (IFN). Then IFN activates the JAK/STAT signaling pathway to up-regulate the expression of TLR3. This cascade reaction is the main mechanism of TLR3-mediated inflammatory response (Latorre et al., 2018). Researchers found that luteolin could reduce the inflammatory response of acute gouty arthritis via down-regulating the TLR/MyD88/NF-κB pathway (Shen et al., 2020). Acacetin not only suppressed the release of pro-inflammatory cytokines IL-6, but also it increased the secretion of anti-inflammatory cytokine IL-10 in cardiomyocyte hypoxia/reoxygenation injury (Wu et al., 2018). Integrin is a family of cell surface receptors that mediates the adhesion of cells to the extracellular matrix (Harburger and Calderwood, 2009). In addition, integrin binds to the extracellular matrix and accumulates in the plasma membrane to form a protein signaling complex–focal adhesion plaque (FA) (Harburger and Calderwood, 2009). Focal adhesion kinase (FAK), one of the downstream components of the focal adhesion complex, is phosphorylated and activated to play an important role in integrin-mediated signaling pathway (Serrels et al., 2015). Cell migration occurs in a variety of physiological and pathological processes such as immunity, inflammation and tumor (Leslie, 2011). FAK binds to proteins containing the Src sequence to form FAK/Src complex. Then FAK/Src complex recruits downstream proteins, such as paxillin to participate in the regulation of cell cycle, adhesion, and metastasis (Cui et al., 2019). Our previous study confirmed that quercetin inhibited the iNOS/FAK-paxillin pathway to limit LPS-induced macrophages migration (Cui et al., 2019). Moreover, kaempferol could predominantly suppress the invasion and migration of renal cancer cells via the downregulation of AKT and FAK pathways (Hung et al., 2017). Our result showed that focal adhesion pathway could be a novel potential targets of SC and PS against pelvic inflammatory disease with dampness-heat stasis syndrome.

In conclusion, the result unambiguously showed that the potential targets and signaling pathway of SC and PS is strongly related to anti-inflammatory effect. Interestingly, the combination of two herbs would strongly inhibit inflammation through different mechanisms, which would be the unique advantage for the treatment.

**FIGURE 11:**
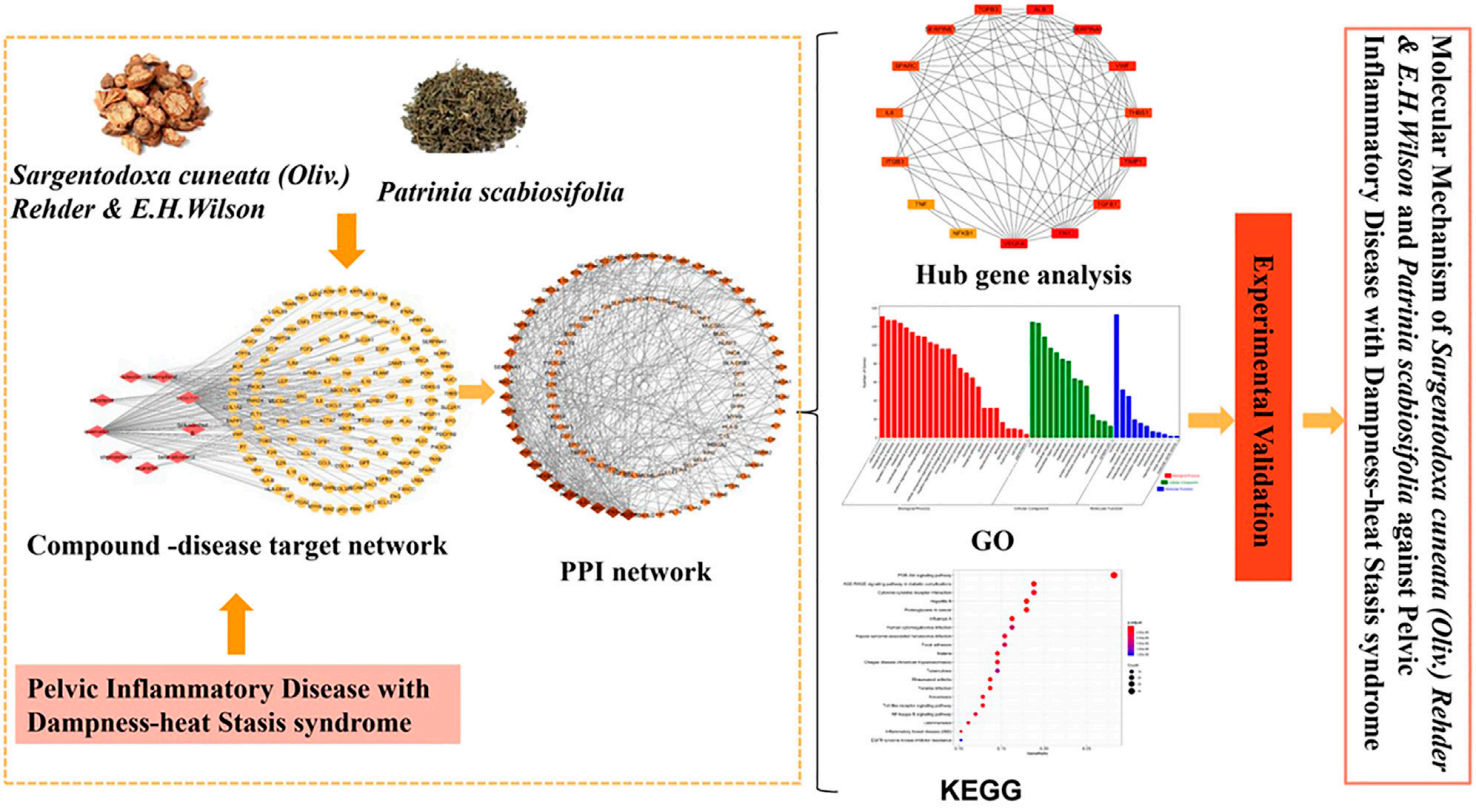
Scheme of Mechanism of SC and PS against Pelvic Inflammatory Disease with Dampness-Heat Stasis syndrome via Network Pharmacology Approach.

In this study, we integrated a variety of method to analysis the molecular mechanism of SC and PS against the pelvic inflammatory disease with dampness-heat stasis syndrome (Figure 11). Different from the previous study, we not only focus on the disease targets, but also combined with the targets of dampness-heat stasis syndrome. The results showed that quercetin, catechin, luteolin and kaempferol were the main components of the anti-inflammatory action of *SC* and *PS.* The main targets include vascular endothelial growth factor A (VEGFA), von willebrand factor (VWF), interleukin 6 (IL6), tumor necrosis factor (TNF) and nuclear transcription factor 1 (NFκB1). They act on the signaling pathways like advanced glycation end products-receptor of advanced glycation end products (AGE-RAGE), focal adhesion (FA), toll-like receptor (TLR) and nuclear transcription factor κB (NF-κB). This study underlies the advantage of the combined application of *SC* and *PS* in the pelvic inflammatory disease with dampness-heat stasis syndrome and provides solid evidence for the molecular mechanism of the treatment. This study provided a theoretical basis for further development of anti-inflammatory drugs for personalized treatment.

## Data Availability Statement

The raw data supporting the conclusions of this article will be made available by the authors, without undue reservation, to any qualified researcher.

## Author Contributions

LH and YC: data curation; writing-original draft preparation. TC and DH: data curation; reviewing and editing. SL: conceptualization; reviewing. SC: conceptualization; supervision; reviewing and editing.

## Funding

This research is supported by National Natural Science Foundation of China (NSFC) (81703969) and Yangzhou University “Qinglan” project (2018).

## Conflict of Interest

The authors declare that the research was conducted in the absence of any commercial or financial relationships that could be constructed as a potential conflict of interest.
